# “Fear of raising the problem without a solution”: a qualitative study of patients’ and healthcare professionals’ views regarding the integration of routine support for physical activity within breast cancer care

**DOI:** 10.1007/s00520-023-08293-2

**Published:** 2024-01-08

**Authors:** K. Gokal, A. J. Daley, C. D. Madigan

**Affiliations:** 1 School of Sport, Exercise and Health Sciences, Loughborough University, Loughborough, Leicestershire LE11 3TU UK; 2 The Centre for Lifestyle Medicine and Behaviour (CLiMB), Loughborough University, Loughborough, Leicestershire LE11 3TU UK

**Keywords:** Physical activity, Breast cancer care, Remote support, Training

## Abstract

**Objective:**

The benefits of physical activity across the cancer continuum for many adult cancers are well established. However, physical activity is yet to be routinely implemented into health services throughout the world. This study aims to explore patients’ and healthcare professionals’ views about integrating conversations and support for physical activity into routine care during treatment for breast cancer.

**Methods:**

Healthcare professionals and patients from across the UK living with or beyond breast cancer were invited to take part in semi-structured interviews that were conducted online. Recruitment for the study was advertised on social media, in cancer support groups and newsletters. Data were analysed using inductive thematic analysis.

**Results:**

Three themes captured perceptions of integrating support for physical activity in routine breast cancer care among 12 health care professionals (who deliver breast cancer care) and 15 patients. Themes between healthcare professionals and patients overlapped, and therefore, combined themes are presented. These were: (1) current practice; (2) implementation in care and (3) training needs.

**Conclusion:**

Many healthcare professionals who offer cancer care are reluctant to raise the topic of physical activity with patients, yet patients have suggested that they would like additional support to be physically active from their medical team. Providing healthcare professionals with education regarding the benefits of physical activity to reduce the risk of recurrence along with evidence based low-cost, remote interventions would allow them to integrate conversations about physical activity within routine cancer care for all patients.

**Supplementary Information:**

The online version contains supplementary material available at 10.1007/s00520-023-08293-2.

## Introduction

The benefits of physical activity for the prevention, management and survival of many adult cancers are well established, as highlighted by multiple systematic reviews [1–3]. Physical activity during and after treatment is safe and acceptable to patients and is endorsed by the World Health Organisation (WHO) and the American College of Sports Medicine (ACSM) [4]. The WHO states that adults living with cancer should avoid inactivity and aim to achieve at least 150 min of moderate-intensity aerobic physical activity per week and encourage muscle-strengthening activities on at least 2 days of the week[5].

Evidence has documented the benefits of participation in physical activity during and after medical treatment for improving psychosocial well-being [6], managing cognitive decline [7], enhancing chemotherapy completion rates [8, 9], reducing the risk of comorbidities such as cardiovascular disease and reducing the risk of recurrence [1, 10]. Despite the confirmed benefits of physical activity for the management of breast cancer, it is not routinely integrated as an essential component of cancer care, and internationally only 6% of oncologists refer their patients to physical activity programmes [11]. Furthermore, 31% of patients in the UK reported being inactive during and after treatment (i.e. completed less than 30 min of moderate-intensity activity a week) [12] and 43% of patients reported becoming less active following their diagnosis [13]. Previous qualitative research with cancer clinicians reported several barriers to the implementation of physical activity advice in routine care, which included a lack of training and knowledge on how to do so [14], time, concerns regarding patient safety [15, 16] and lack of accessible patient referral pathways to exercise/physical activity specialists [17, 18]. However, further research is required to explore facilitators to the integration of physical activity into the patient pathway. Supporting patients to change their health behaviours and reduce the burden of disease is becoming a greater feature within the role of healthcare professionals (HCPs) through initiatives such as Making Every Contact Count.

Therefore, this study aims to explore patients’ and HCPs’ views and experiences of participating in conversations offering support for physical activity in routine care during treatment for breast cancer. The study will contribute towards the development of future health service policy and implementation of support for cancer patients to engage in a physically active lifestyle to manage treatment-related side effects and reduce their risk of recurrence.

## Methods

### Design

A qualitative, semi-structured interview study was conducted with breast cancer patients and HCPs. The study is reported in line with the COREQ guidelines (see supplementary document). Ethical approval was granted by the Loughborough University Research Ethics Committee (Ref: 2022–5729-10266). Data collection for this study took place between July and November 2022.

### Participants and recruitment

#### Healthcare professionals

HCPs providing cancer care in the UK (i.e., including oncologists, surgeons and cancer nurses) were invited to take part in an online semi-structured interview. Snowballing sampling methods were used to recruit across the UK via special interest groups, mailing lists and social media.

#### Patients

Patients who were undergoing treatment or had completed treatment for breast cancer in the UK were invited to take part using snowball sampling methods. The study was advertised via social media, cancer support groups and newsletters. Initially, we aimed to recruit patients who had received their cancer diagnosis within three years of the interview. However, after the initial few interviews, it became apparent that those diagnosed within the three years experienced a change in routine cancer care due to disruptions caused by the COVID-19 pandemic. Therefore, a broad inclusion criterion was adopted where all breast cancer patients, regardless of time elapsed since diagnosis, were eligible to take part in this study.

### Data collection

Informed consent was taken verbally or via email prior to the interview which took place via MS Teams or telephone, based on participants’ preference. The duration of the interviews ranged between 20 and 50 min. Only the participant and the interviewer (KG) were present for the interviews and there was no prior relationship.

Two semi-structured interview schedules (one for HCPs and another for patients) were developed by KG and AJD in line with the aims of the study. Interviews focused on understanding perceptions of physical activity, potential benefits, current practice for discussing physical activity in cancer care and facilitators to routine conversations within consultations. Demographic characteristics and current levels of physical activity using the exercise vital signs questionnaire [19] were gathered from patients to gain insight into their typical levels of physical activity.

All interviews were conducted by KG, audio-recorded and transcribed verbatim. Reflective notes were taken after each interview to summarise key themes and to inform iterations to the interview schedule where necessary. KG is a female senior research fellow with a PhD and expertise in mixed methods research with a range of clinical and non-clinical populations including those with breast cancer. KG is positive about promoting physical activity for the prevention and management of cancer and recognised that this could bias interpretation of the results; thus, CDM (who does not research cancer rehabilitation) provided peer-to-peer debriefing [20] when analysing the results.

### Data analysis

**Fig. 1:**
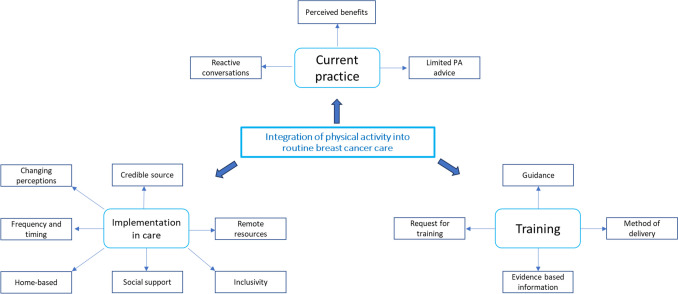
Thematic map

All transcripts were anonymised and uploaded to NVivo 12. Inductive thematic analysis was followed to identify and analyse reporting patterns within the data. Coding and analysis were conducted by KG in parallel with data collection. Interviews were stopped when it was deemed that no new themes were occurring. Interviews with patients and HCPs were analysed separately but were presented together as themes overlapped. Emerging themes were discussed with AJD and CDM regularly throughout data analysis and were supported by the reflective notes. Emerging themes were reviewed by KG and CDM and thematic mapping (see Fig. 1) was used to ensure an accurate representation of the data to move beyond description and support the development of the theme. Minor modifications were made to the titles of themes and subthemes during this iterative process, and no substantial disagreements in themes or subthemes were noted.

## Results

### Participant characteristics

Fifty-four patients and twenty-one HCPs expressed an interest in participating in this study and 26 consented: 11 HCPs (Table 1) and 15 patients (Table 2). All patients were female and had received a diagnosis of breast cancer in the UK within 6 months to 10 years of the date of the interviews. Patients had a mean age of 59 years (SD 9.6), most were employed (40%), Caucasian (90%) and received their treatment exclusively via the National Health Service (NHS) (67%). On average, patients self-reported that they completed 210 min (SD 132.9) of physical activity per week. Most HCPs were female (91%), with experience ranging between two and nineteen years and included one surgeon, five oncologists and five cancer nurse specialists across seven NHS healthcare trusts in the UK.

**Table 1:** Healthcare professional demographic characteristics

ID	Role	Years of service	Aware of PA guidelines	Routinely promote PA
HCP1	Clinical oncologist	3	Yes	yes
HCP2	Chemotherapy nurse	2	no	no
HCP3	Consultant chemotherapy nurse	11	no	yes
HCP4	Lead oncology nurse	4	no	no
HCP5	Lead cancer clinical nurse specialist	19	no	no
HCP6	Surgeon	Missing	Missing	no
HCP7	Oncologist	15	yes	no
HCP8	Oncologist	17	yes	no
HCP9	Advanced nurse practitioner	3	no	no
HCP10	Consultant clinical oncologist	19	no	no
HCP11	Consultant medical oncologist	3	no	no

**Table 2:** Patient demographic characteristics

ID	Age (years)	Employment status	Ethnicity	BC grade	Time since diagnosis (years)	Adj/Neo Adj chemotherapy	NHS/private (P) healthcare	Location of treatment	Total mins MVPA	PA discussion with clinician (Y/N)	Awareness of cancer and PA guidelines (Y/N)
P001	39	Self-employed	White	2	2	Adjuvant	NHS	Bath	450	Y	N
P002	51	Employed	White	3	13	Adjuvant	NHS	Leicester	360	N	Y
P003	51	Self-employed	White	2	10	Neo-adjuvant	NHS	Slough	0	N	N
P004	64	Retired	White	3	5	Neo-adjuvant	NHS	Leicester	60	N	N
P005	62	Retired	White	3	5	Adjuvant	NHS + P	Leicester	260	N	N
P006	50	Sick leave	White	3	< 1	Neo-adjuvant	NHS + P	Glasgow	135	Y	N
P007	35	Sick leave	Black African	3	< 1	Neo-adjuvant	P	London	360	Y	Y
P008	47	Employed	White	Missing	1	NA	NHS	Plymouth	280	N	N
P009	46	Employed	White	2	< 1	Neo-adjuvant	NHS	Newcastle	240	N	Y
P010	36	Employed	White	2	2	Adjuvant	NHS	Kent	315	N	N
P011	55	Unemployed	White	Missing	< 1	Adjuvant	NHS	Leicester	90	N	N
P012	60	Employed	White	2	2	Adjuvant	NHS	Leicester	310	N	N
P013	44	Self- employed	White	2	< 1	Adjuvant	P	Milton Keynes	90	Y	Y
P014	42	Employed	White	3	< 1	Adjuvant	P	Milton Keynes	Missing	Y	N
P015	63	Unemployed	White	2	< 1	neo-adjuvant	NHS	Bath	120	Y	N

### Themes

Themes between HCPs and patients were very similar; therefore, combined themes exploring current practice and the perceptions of integrating conversations about physical activity in routine cancer care are presented. Three themes emerged (with 14 subthemes); current practice, implementation in care and training (see Fig. 1).

### Theme 1: Current practice

#### Limited physical activity advice

Most patients and HCPs were not aware of the cancer-specific physical activity guidelines and most patients did not recall receiving information about the benefits of being physically active during or after treatment for cancer (Table 3). Those who recalled conversations about physical activity were patients who received their treatment via private healthcare (discussed further below) and only two NHS patients (from the same Trust) recalled being asked about their physical activity levels by their medical team.

**Table 3:** Supplementary data

Theme	Sub-theme	Quotations
Current practice	*Limited PA advice*	**HCP5**: I think it’s probably down to individual teams if they’ve got people in there who actually. You know, believe in the benefit of physical activity **HCP2**: Where I work, obviously we promote it by like asking how people are getting on, like if they’re feeling fatigued. If they’re able to, like do normal things that they might do if they were like avid gym goers or runners or something. But then it’s just kind of dismissed
	*Reactive conversations*	**HCP5**: I do talk to them about it, but it’s probably not something that automatically comes into conversations unless that I can either see a need or people are expressing. “Ohh really wish I can get back to the gym”, that type of thing **HCP9**: Yeah, I guess if it comes up in conversation or patients say this troubling about the weight or things like that then then I would talk about it **HCP 6:** I think I would be honest and just say its more than likely if a patient mentions it, then I will do it, but I just, if you’re already giving them a lot of information and as well where I just think it’s almost the sort of thing that you could drip feed in later
	*Perceived benefits in:* NHS Private care:	**P01:** I know for my mental and physical health how important keeping active was and like I say, the fatigue you kind of think I just need to rest, but actually it helped me manage my fatigue a lot more **HCP10**: So I understand it has an important role to play because and not just in terms of people’s sort of emotional, mental, psychological well-being, but their physical well-being as well, because then I know that a little bit of exercise can help boost and their energy even in people who are quite fatigued and from their disease or their treatments… **HCP1**: during treatment and after treatment is very beneficial, so during treatment it helps in a uh, to battle fatigue after treatment. It helps to prevent recurrence in or things like that **P013**: And he absolutely drummed into me heart, heart, heart. Go look after it. Through chemo, you gotta keep moving. You gotta keep heart rate up and you know, you’ve got to keep exercising and how important it is to protect your heart through chemo through exercise
Implementation in care	*Remote resources*	**P10:** it’s a lot to take in, but I think if you’re given all the leaflets and stuff, and then you can obviously take them home and discuss them with friends and relatives, that’s probably a really good time as well. I think definitely leaflets, something written down because you’re taking so much in all the time. And certainly when you’re really stressed, it’s very difficult to remember everything from an appointment **P015**: But I mean with the proliferation of apps, maybe an app with targets to reach each day would be helpful **HCP1**: but would be nice to have something to give them. You know something that can be in clinic and just used to encourage them
	*Home-based*	**P10:** people that are going through treatment are more likely to spend more time at home, rather than out in a class or something, just because of energy levels
	*Credible source*	**P11:** But knowing that it’s research based is important for me because you have got common sense because goes out the window because I’ve never been ill **P05:** if they’re saying you should be doing this, you know, they know what they’re talking about sort of thing! So yeah, I would … that would motivate me as well, yeah **P15**: In an ideal world, I think it should come from a medical practitioner! Because it would give it more credibility
	*Inclusivity*	**P09**: I definitely think the advice should be for everyone to maintain any and you know, and if possible improve obviously very active people might not be able to improve, but with the idea to help people maintain though, **HCP6:** I would say that I would think that there’s probably a way in which they could all be physically active **HCP11**: even people who are wheelchair bound you know? Or nursing homes? I know they often have, you know physical exercise programs where they can do stretches and arm things and give various different things with their legs and rotate their ankles and bring their knees up to their chests and that type of thing so
	*Changing perceptions*	**P15:** I was constantly told by family, don’t overdo it, don’t go too far, you know … and sort of fighting that … in a way, I mean there was some encouragement to go for a walk but not to go more than round the block at times you know and … so any kind of information in printed form that I could show them would have been helpful **P03:** The business about people saying, oh, we should conserve your energy and you shouldn’t exercise while you’re on treatment. I think would be useful to have some maybe. Maybe if they’ve got some common myths busting them as well, that would be. I think that would be useful **HCP5**:. I’ve been sitting with patient who have been given a diagnosis, and I’ve had sort of the family member saying. Right, OK, you go home, we’ll go home and you just sit down and you won’t have to do anything. And we’ll get people coming in to do this and that. Hold on a moment. Yeah, that sounds very lovely and very supportive. However, we want you to keep up and about, you know
	*Frequency and timing of conversations*	**P05:** but it might be something that might need to be given more than once, if you know what I mean because you might be given that information on your pre-op visit but you might not be in a position that you feel well enough to do anything for a while and then you might forget about it or you know …So I suppose you’d need some follow-up between each to see … you know it could be part of the check-in couldn’t it **P06:** I think throughout because I experienced different things at different points of my treatment **P11**: definitely when you’re embarking on your active treatment at that earlier point to make a connection and maybe halfway through because then you’re sort of a bit more chemo savvy **P14**: it needs to be driven home again, you know, or it can be driven home again at that point if it’s in your head like two or three weeks before you start chemo, then you’re already thinking about it when you are OK well, you know I can I can plan for this **HCP8:** And I think it’s probably a repeated thing for a number of reasons. So inevitably there needs to be a you know, followed up and tracked going along. So I think that there are perhaps key nodal points, when it it’s really helpful to introduce it
	*Social support*	**P13:** I think if you could get like buddies like a buddy system. So say someone like me who’s been through it, who has done the exercise **P02**: It needs a bit more time and sympathy, not someone that’s going, oh come on just get on with it, they need someone that’s been through it, understands how hard it is and has patience and understanding, I think that’s real key
Training	*Requests for training*	**HCP1**: So I think that’s where the training or teaching would be helpful to know when to integrate it to patients and how to integrate it **HCP 11**: And training session would be good **HCP4**: I think it has, it’s more of a topic now that people are aware of but a training package and some advice and what sort of and how you would assess and what physical activity is appropriate **HCP6:** And up-to-date information. Basically, for the clinicians that would be involved in it, and wouldn’t there’s an educational aspect of things that would be needed amongst the staff that were going to be delivering **HCP9:** kind of like education in a team and to know what words you’re going to use to make sure people understand it and what you’re actually asking of them and what you’re suggesting of them
	*Evidence-based info*	**HCP11:** If there is data there that I can come can see, then I think that would help **HCP8:** I think some of the key evidence base that supports it,’cause you know, we’re very evidence driven in oncology **HCP5**: I think the information with the evidence behind it
	*Guidance*	**HCP11**: And in terms of physical activity, what types of physical activity are safe? **HCP10:** given them some pointers, the types of exercises, the duration of exercise. So getting that balance between doing the physical activity where it’s going to boost their energy and help them with their treatment but not so much that they’re going to be exhausted and then not be able to carry on with their treatment **HCP5**: I think it’s a lot of times we’re gonna have to be doing really small steps with patients. So you know how, what are the ways to if you’ve got someone who’s completely inactive, who really and we do have completely inactive people, how do you start off, how do you, how do you get that motivation? How do you get that? What are the, what are the key sort of like terms or words to use that you know, what’s the health benefit?

#### Reactive conversations

When physical activity was discussed by HCPs with patients, conversations were “*usually a reactive discussion*” (HCP11) in response to patients first raising the topic and ‘…*pushing it more”* (P01) or when “…*it comes up in conversation…then then I would talk about it”* (HCP9). HCPs noted that they tended to discuss physical activity with patients who had a personal interest in the activity and were seeking permission to remain active through their cancer treatment. HCPs avoided raising the topic of physical activity as they did not have the resources or infrastructure to provide their patients with sufficient support: “*At the moment we have no solution, so it’s actually it makes us feel bad to raise a problem when you can’t offer the patient support and solutions, so I suspect that’s why it’s not being raised at all”* (HCP7).

#### Perceived benefits- in the NHS

The perceived benefits of physical activity generally were understood by most patients, with very little knowledge expressed about the specific benefits physical activity can have on cancer treatment-related outcomes. Patients referred to the potential benefits of activity for their mental and physical health, fatigue or sleep: “*We’d have a walk, and just doing that though was lovely because mentally it did me a lot of good”* (P05) and “*you kind of think I just need to rest, but actually it helped me manage my fatigue a lot more”* (P01). Similarly, when HCPs spoke about the benefits of physical activity, most referred to psychosocial well-being as opposed to managing cancer treatment-related outcomes. Only one HCP mentioned that physical activity may be beneficial for reducing patients’ risk of recurrence.

#### Perceived benefits- in private care

A subsample of patients (*n* = 5) who received private treatment were offered guidance from their healthcare providers and given practical support, enabling them to be active during treatment. When discussing the importance of physical activity, patients treated privately referred to the role of activity in improving their prognosis and survival following treatment, in addition to the psychosocial benefits. For example, “*We know as well from research that exercise after cancer can reduce your risk of it coming back by 50%”* (P013), and another patient discussed the importance of resistance-based training: *“It’s really good for your bone density…you know chemo knackers your bone density and it can bring on osteoporosis”* (P14).

### Theme 2: Implementation of care

The second theme includes seven subthemes that explore the perceived components necessary for the successful implementation of routine conversations about physical activity within breast cancer care. The subthemes identified through joint analysis of data include (1) remote resources, (2) home-based activity, (3) credible source (4) inclusivity, (5) changing perceptions (6) frequency and timing of conversations and (7) social support.

#### Remote resources

Patients are often overwhelmed with information provided in medical consultations, and therefore, having materials that they could refer to in their own time with family or friends was perceived as important. Both patients and HCPs mentioned the potential benefits of promoting activity using a mobile phone application to encourage engagement with the information and self-monitoring of behaviours: “*You could make an app, could make that interactive like patients could log their activities and then see in a month’s time or in a during a weekend which they did like”* (P08). We could say, ***“****Here’s an app that would potentially give you some information about what you can and can’t do…I think that would be really great actually, because I don’t think tagging on at the end of a booklet or giving them another full booklet about it would necessarily work”* (HCP6).

#### Home-based

Participants from both groups expressed concern regarding structured exercise and commented on the importance of flexibility in the type and location of physical activity promoted. Breast cancer patients often spend a lot of time attending hospital visits, which can be both time-consuming and tiring. Therefore, additional scheduled or supervised classes were viewed as creating barriers to attendance. Home-based, flexible activities were preferred by most patients. Similarly, attendance at classes located in public spaces were less appealing due to the increased risk of infection during treatment. Lastly, home-based activity was perceived as less daunting as it would minimise the need for interaction with other people at a time when patients have reduced self-esteem and additional concerns regarding their body image and hair loss: “*If it was in their own home. It was very safe for, you know, and they didn’t have to be in a place where people could see them…and they didn’t actually have to go anywhere to do it”* (HCP5).

#### Credible source

Patients expressed preferences for having evidence-based information about physical activity and guidance from a trustworthy source at a time when they are vulnerable: “*If you can say to them this is evidence based, this is how much you know it decreases your fatigue*” (P13). Likewise, HCPs were conscious that patients trusted the information they provided and were more likely to perform the behaviour when introduced by a professional: *“…if there was an endorsement from a healthcare professional it was much more likely that patients were interested in participating”* (HCP6).

#### Inclusivity

When specifically asked, there was a clear indication that patients and HCPs believed that *“the advice should be for everyone”* (P09), and there was no need for additional structured physical activity assessments prior to its promotion as “…*there’s probably a way in which they could all be physically active…*..*even if its just small changes*” (HCP6). While also acknowledging that physical activity should be tailored to each individuals’ abilities. “*It’ll have to be sort of tailor made to their abilities”* (HCP10).

#### Changing perceptions

Both patients and HCPs mentioned the importance of engaging with and changing the perceptions of loved ones, who often show their support by asking patients to rest. Patients expressed that they were “*constantly told by my family, don’t overdo it*” (P15) or *“conserve your energy and you shouldn’t exercise while you’re on treatment*” (P03), and therefore, requested materials for family members outlining “*any kind of information in printed form that I could show them would have been helpful*” (P15).

#### Frequency and timing of conversations

Patients and HCPs both commented on a preference for multiple conversations about the importance of physical activity *“might need to be given more than once”* (P05) throughout patients’ treatment pathway. It was felt that patients should be made aware of the importance of physical activity before they begin treatment while acknowledging that they may engage in physical activity at different time points during their treatment.

#### Social support

Patients commented that the ability to connect with others with shared experiences could facilitate physical activity: “*If you had people on the app and you know, they can become friends during treatment and then follow each other on it and then encourage each other”* (P14).

### Theme 3: Training for healthcare professionals

Iterations were made to the HCP interview schedule based on the recurring theme of additional training to support conversations about physical activity within routine cancer care. Analysis revealed four subthemes including the following: (1) requests for training, (2) evidence-based information, (3) guidance and (4) method of delivery.

#### Requests for training

HCPs expressed an interest and need for further training to confidently raise awareness and deliver brief conversations about physical activity. For example, “*Maybe just a kind of a training package and you know, I think there’s some nurses out there probably still don’t really know how important it is, so really kind of selling it*” (HCP4).

#### Evidence-based materials

Credible information to inform brief conversations about physical activity was also requested; for example, “*I think some of the key evidence base that supports it, ‘cause you know, we’re very evidence driven in oncology*” (HCP8).

#### Guidance

The need for guidance regarding the type and duration of physical activity, as well as appropriate terminology and timing to support the integration of brief conversations about physical activity, was also noted by several HCPs. HCP1: *“I think it’s how to incorporate it and when to incorporate it. So I think that’s where the training or teaching would be helpful to know when to integrate it to patients and how to integrate it.”*

#### Method of delivery

There was a preference for a training module that could be delivered remotely as opposed to the need to attend a scheduled event in person. HCP11: *“If you’ve got to make a physical trip somewhere, I think it just adds travel time to an event, then it can mean that makes the difference between something being feasible or not. So I think a remote.”*

## Discussion

### Summary of findings

This study provides useful insight and practical guidance to support the integration of physical activity into the pathway for all patients with breast cancer. Findings confirm that physical activity is not routinely discussed with all patients who receive treatment for breast cancer in the UK. HCPs are reluctant to discuss physical activity, yet commented that they would be comfortable promoting it, following training, as well as access to clear, evidence-based resources to support such conversations with their patients. Both patients and HCPs raised concerns about the logistics and safety of attending group based, scheduled activity sessions in public gyms and favoured home-based, self-managed activity programmes.

### Interpretation of findings

Most patients and HCPs in our study were unaware of the physical activity guidelines for those living with and beyond a cancer diagnosis, and the recommendations were not routinely discussed in consultations. Conversations with patients about physical activity were reactive, and therefore, breast cancer patients who are physically inactive and most likely to benefit from receiving support [22] in their consultations are currently not supported to become more active. Given the side effects of cancer treatment and that most women diagnosed with breast cancer are aged 50 years or older, it is particularly important that all women are advised to engage in strength-based physical activity twice per week in line with guidance [5] to reduce their risk of osteoporosis and maintain muscle mass [23, 24]. Furthermore, patients who were actively educated about the benefits of physical activity were those who received private healthcare, raising questions about further health inequalities for this population. Providing guidance for all patients to raise awareness and improve knowledge regarding all the health benefits that physical activity provides is critical for reducing health inequalities and reducing the risk of recurrence.

### Requests for physical activity guidance in routine care

HCPs were reluctant to raise the topic of physical activity without having adequate knowledge and resources to provide a solution to reducing inactivity among their patients. Participants from this study echoed similar barriers to promoting physical activity in cancer care as noted in previous studies [14, 17, 18]. Yet both patients and HCPs commented on the necessity and importance of conversations about physical activity for improving health outcomes within the patient pathway. Our findings emphasise the willingness of patients and HCPs to integrate physical activity into routine cancer care. These findings support the ACSM’s (5) call to action, highlighting the importance of implementing exercise as medicine within oncology. Findings echo the requirement for oncology clinicians, who are recognised as trustworthy and credible sources of information among patients, to advise patients to increase their physical activity.

### Integration of physical activity in routine care

HCPs in our study believed that all patients treated with breast cancer could engage in physical activity and acknowledged that small bouts of activity may be more realistic for the most inactive, in line with current WHO recommendations [25]. HCPs commented that all patients could engage in some form of physical activity regardless of their abilities, suggesting that prior assessment of physical activity was not essential for its promotion within the cancer pathway for those treated with breast cancer. In line with previous evidence, brief conversations and multiple contacts from HCPs to support health behaviour change [26] were deemed essential to prevent overloading patients with information.

### Remote, self-managed resources

Previous literature noted the lack of time in consultations as a key barrier to implementation [14] but was not expressed as a concern by HCPs in our study. However, participants commented on the facilitators of physical activity promotion through methods that are brief and self-efficient i.e. referral toward evidence-based resources such as a mobile application, which require minimal clinical burden. Emphasis was placed on providing patients with the ability to self-manage their activity as opposed to the need to attend structured rehabilitation. Self-managed, home-based activities were preferred due to their flexibility and potential to minimise body-image concerns associated with attending public spaces and reducing the risk of infections.

### Training

Consistent with other studies, most HCPs were unaware of the cancer-specific physical activity guidelines and therefore identified training as an essential element to the successful integration of physical activity. Requests were made for remote training modules, which outline the evidence and provide practical guidance and access to remote resources to facilitate effective signposting.

### Implications of work

Our findings indicate that brief conversations, delivered by a credible source and followed up at multiple stages of the treatment pathway promoting physical activity, should be integrated into the treatment pathways for all patients treated with breast cancer. The provision of remote resources would provide HCPs with the confidence to promote physical activity whilst acting as a credible resource for patients living with and beyond cancer. Mobile applications are scalable, with the potential to reduce health inequalities while providing patients with the resources to promote self-management of home-based physical activity, which are perceived as facilitators to behaviour change during cancer treatment.

### Strengths and limitations

The in-depth interview approach with a broad sample of patients and HCPs across multiple NHS Trusts and private healthcare settings are the strengths of the current study. Furthermore, the range of expertise in the study team is a strength as it allowed the critical exploration of current practice and identification of optimum methods for integrating conversations about physical activity into routine cancer care. Our patient sample differed across a range of demographic characteristics, yet the majority self-reported above-average levels of physical activity; therefore, our sample may not represent patients who are physically inactive. While overestimation of self-reported physical activity is typical, above-average levels of physical activity among our sample of patients may be due to selection bias and is a limitation of the study. Therefore, findings should be interpreted with caution, and future research should place further emphasis on engaging with inactive patients.

### Conclusion

Many HCPs who offer cancer care are reluctant to raise the topic of participation in physical activity, yet patients would welcome discussions. Providing HCPs with education regarding the benefits of physical activity along with evidence-based, low-cost, remote interventions would allow them to integrate conversations about physical activity within routine breast cancer care for all patients, ultimately improving treatment outcomes and reducing their risk of recurrence.

### Supplementary Information

Below is the link to the electronic supplementary material.

Supplementary file1 (DOCX 24 KB)
