# Supplementary material for: “Fear of raising the problem without a solution”: a qualitative study of patients’ and healthcare professionals’ views regarding the integration of routine support for physical activity within breast cancer care
Source: Support Care Cancer. 2024 Jan 8;32(1):87. doi: 10.1007/s00520-023-08293-2 (PMC10771998; doi:10.1007/s00520-023-08293-2)
Supplement: Supplementary file 1 — Supplementary file1 (DOCX 24 KB) [file 520_2023_8293_MOESM1_ESM.docx]

**Supplementary document: COREQ checklist**

| Consolidated criteria for reporting qualitative studies (COREQ): 32-item checklist | | | | | Page Number | |
| --- | --- | --- | --- | --- | --- | --- |
| Domain 1: Research team and reflexivity | | | | | | |
| Personal Characteristics | | |  | | |  |
| 1. Interviewer/facilitator | Which author/s conducted the interview or focus group? | | | | | 4 |
| 1. Credentials | What were the researcher’s credentials? E.g. PhD, MD | | | | | 4 |
| 1. Occupation | What was their occupation at the time of the study? | | | | | 4 |
| 1. Gender | Was the researcher male or female? | | | | | 4 |
| 1. Experience and training | What experience or training did the researcher have? | | | | | 4 |
| Relationship with participants |  | |  | | |  |
| 1. Relationship established | Was a relationship established prior to study commencement? |  | | 4 | | |
| 1. Participant knowledge of the interviewer | What did the participants know about the researcher? e.g. personal goals, reasons for doing the research |  | | 4 | | |
| 1. Interviewer characteristics | What characteristics were reported about the interviewer/facilitator? e.g. Bias, assumptions, reasons and interests in the research topic |  | | 4 | | |
| Domain 2: study design | | | | | | |
| Theoretical framework | | |  | | |  |
| 1. Methodological orientation and Theory | What methodological orientation was stated to underpin the study? e.g. grounded theory, discourse analysis, ethnography, phenomenology, content analysis. | | | | | 4 |
| Participant selection | | |  | | |  |
| 1. Sampling | How were participants selected? e.g. purposive, convenience, consecutive, snowball | | | | | 3 |
| 1. Method of approach | How were participants approached? e.g. face-to-face, telephone, mail, email | | | | | 3 |
| 1. Sample size | How many participants were in the study? | |  | | | 5 |
| 1. Non-participation | How many people refused to participate or dropped out? Reasons? | | | | | 5 |
| Setting | | |  | | |  |
| 1. Setting of data collection | Where was the data collected? e.g. home, clinic, workplace | |  | | | 4 |
| 1. Presence of non-participants | Was anyone else present besides the participants and researchers? | | | | | 4 |
| 1. Description of sample | What are the important characteristics of the sample? e.g. demographic data, date | | | | | Table 1, Table 2 |
| Data collection | | |  | | |  |
| 1. Interview guide | Were questions, prompts, guides provided by the authors? Was it pilot tested? | | | | | 4 |
| 1. Repeat interviews | Were repeat interviews carried out? If yes, how many? | |  | | | - |
| 1. Audio/visual recording | Did the research use audio or visual recording to collect the data? | | | | | 4 |
| 1. Field notes | Were field notes made during and/or after the interview or focus group? | | | | | 4 |
| 1. Duration | What was the duration of the interviews or focus group? | |  | | | 4 |
| 1. Data saturation | Was data saturation discussed? | |  | | | 4 |
| 1. Transcripts returned | Were transcripts returned to participants for comment and/or correction? | | | | | - |
| Domain 3: analysis and findings | | | | | | |
| Data analysis | | |  | | |  |
| 1. Number of data coders | How many data coders coded the data? | |  | | | 4 |
| 1. Description of the coding tree | Did authors provide a description of the coding tree? | |  | | | 4 |
| 1. Derivation of themes | Were themes identified in advance or derived from the data? | |  | | | 4 |
| 1. Software | What software, if applicable, was used to manage the data? | |  | | | 4 |
| 1. Participant checking | Did participants provide feedback on the findings? | |  | | | - |
| Reporting | | |  | | |  |
| 1. Quotations presented | Were participant quotations presented to illustrate the themes / findings? Was each quotation identified? e.g. participant number | | | | | 5-9 |
| 1. Data and findings consistent | Was there consistency between the data presented and the findings? | |  | | | 10 |
| 1. Clarity of major themes | Were major themes clearly presented in the findings? | |  | | | 5-9 additional files |
| 1. Clarity of minor themes | Is there a description of diverse cases or discussion of minor themes? | | | | | 5-9 additional files |

**HCP semi-structured interview schedule**

|  |  |
| --- | --- |
| **Introduction** | Confirm location, job role speciality, length of service in that role  Do you do much physical activity yourself? |
| **PA** | What is your understanding regarding the role of physical activity in cancer care |
|  | Do you know what the cancer specific physical activity guidelines are for patients during treatment? |
|  | Do you discuss physical activity with your patients at any stage of their treatment? Or after they have completed treatment? |
| If ‘yes’ | What information about physical activity do you provide your patients, if any?  Do you discuss physical activity with all your patients? Explore.  At what point in their treatment schedule is it discussed, and do you provide them with any additional resources? (Leaflets or signposting).  Do you find many patients seek advice about being physically active?  Is this conversation followed up with the patient at a later date?  How do you think patients perceive the conversation about physical activity? |
| If ‘no’ | Is there any particular reason you do not discuss physical activity with your patients? Explore |
|  | Are you aware of any other members of staff (ie. oncologists, nurse, charity reps) that discuss physical activity with patients at any stage of their treatment in secondary care? |
|  | What would need to happen for you to mention physical activity in your consultations/ care during treatment? |
|  | Do you know of any future plans/ policies in place within your institution (NHS more generally) regarding the integration of physical activity into cancer care both during or after treatment? |
| **Oncologists/ nurse role** | Do you feel discussing physical activity is part of your role as a HCP? |
|  | Do you provide information about other health behaviours, like diet, smoking, immunisations etc? Explore |
| **Implementation** | From an operational perspective, when would be the most practical time to introduce PA?  Would you feel confident bringing up PA with all patients? Explore this further. |
| Training | What would you like to gain from this training and what topics would you like to see included?  Explore logistics |

**Patient semi-structured interview schedule**

|  |  |
| --- | --- |
| **Introduction** | 1. Age 2. What is your current employment status 3. What is your ethnicity (i.e. White, Black African, Black Caribbean, Black other, Indian, Pakistani, Bangladeshi, Chinese, other Asian, mixed) 4. Confirm Cancer type and if primary or secondary cancer 5. Length of time since diagnosis 6. What grade was your breast cancer? 7. Did you receive care via the NHS or privately? 8. Location/ city or hospital where treatment was received 9. Treatment received |
| **PA (Vital signs)** | -On average, how many days per week do you engage in moderate to vigorous physical activity (like a brisk walk)? _____ days  -On average, how many minutes do you engage in physical activity at this level? _____ minutes Total minutes per week of physical activity (multiply #1 by #2) _____ minutes per week  Would you say that your activity levels altered after your diagnosis? Explore |
| **PA benefits/ prescription** | What is your understanding regarding the role of physical activity in cancer care (specifically breast)?  Are you aware of how much activity is recommended for those who have received a diagnosis of breast cancer? During treatment and beyond? |
| **PA support** | -Did anyone providing you with medical care during your cancer treatment at the hospital discuss physical activity with you at any stage of your treatment? Or after you completed treatment? i.e. oncologist, chemo nurse, surgeon etc |
| If ‘yes’ | **-What** information about physical activity did you receive? Was it verbal or were you provided with any additional resources?  **-Who** did you receive this information from?  -**When** did you receive the information about physical activity?  explore further |
| If ‘no’ | -Did you receive information about physical activity before or after treatment from any other sources (outside of their medical team)?  -If you were to receive support or encouragement about physical activity during your cancer treatment, **who** would you like to receive the support from?  -**How** would you like to receive encouragement/support regarding the importance of being physically active during treatment?  -**What** would (have) encourage you to be active during treatment? |
| **Other health behaviours** | Did you receive information about other health behaviours, like diet, smoking, immunisations etc by your medical team (oncologist, chemo nurse etc)? Explore |
| Resources | What information/ resources would have helped you to be more active during treatment? |
